# Verification of gait analysis method fusing camera-based pose estimation and an IMU sensor in various gait conditions

**DOI:** 10.1038/s41598-022-22246-5

**Published:** 2022-10-21

**Authors:** Masataka Yamamoto, Koji Shimatani, Yuto Ishige, Hiroshi Takemura

**Affiliations:** 1 Faculty of Science and Technology, Tokyo University of Science, Noda, 278-8510 Japan; 2 Graduate School of Advanced Science and Engineering, Hiroshima University, Higashi-Hiroshima, 739-8527 Japan; 3 Faculty of Health and Welfare, Prefectural University of Hiroshima, Mihara, 723-0053 Japan

**Keywords:** Rehabilitation, Biomedical engineering

## Abstract

A markerless gait analysis system can measure useful gait metrics to determine effective clinical treatment. Although this gait analysis system does not require a large space, several markers, or time constraints, it inaccurately measure lower limb joint kinematics during gait. In particular, it has a substantial ankle joint angle error. In this study, we investigated the markerless gait analysis method capability using single RGB camera-based pose estimation by OpenPose (OP) and an inertial measurement unit (IMU) sensor on the foot segment to measure ankle joint kinematics under various gait conditions. Sixteen healthy young adult males participated in the study. We compared temporo-spatial parameters and lower limb joint angles during four gait conditions with varying gait speeds and foot progression angles. These were measured by optoelectronic motion capture, markerless gait analysis method using OP, and proposed method using OP and IMU. We found that the proposed method using OP and an IMU significantly decreased the mean absolute errors of peak ankle joint angles compared with OP in the four gait conditions. The proposed method has the potential to measure temporo-spatial gait parameters and lower limb joint angles, including ankle angles, in various gait conditions as a clinical settings gait assessment tool.

## Introduction

Gait metrics such as gait speed, stride length, and joint kinematics, are crucial assessment parameters for persons with gait disability in clinical settings. Temporo-spatial and kinematic parameters of gait have been used to assess the treatment effect in neurological disorders^1,2^ and to predict fall risk in elderly individuals^3^. These metrics provide valuable information for planning physiotherapy treatments and determining the treatment effects. Currently, optoelectronic marker-based three-dimensional motion capture (3DMC) is used as a typical measurement tool for clinical gait analysis and can accurately measure gait metrics. The optoelectronic 3DMC system has reliability and repeatability of gait metrics^4,5^. Although the 3DMC accurately measure gait metrics, it is difficult to employ it in a clinical setting because of economic and time constraints^6^. Furthermore, it requires a large space and technical skills for measurement. Inertial measurement unit (IMU)-based motion capture system are also used as an alternative method to 3DMC^7^. However, this system requires the attachment of many IMU sensors to human body segments. Camera-based markerless motion capture system using human pose estimation algorithms are used to measure human gait as an alternative method to 3DMC. Microsoft Kinect, which comprises RGB cameras equipped with a depth sensor, can measure gait without reflective markers. Kinect can measure temporo-spatial parameters, such as gait speed, step time, and step length with high reliability^8–10^. Furthermore, it is used as an exergaming aid for prefrail and frail individuals, and as a cognitive function assessment tool for measuring dual-task gait^11,12^. However, previous studies have reported that depth cameras cannot accurately measure kinematic parameters, such as lower limb joint angles, compared with optoelectronic 3DMC system^8,10,13^. In addition, markerless motion capture systems using depth sensors cannot easily measure fast joint movements because of the sampling rate. The sampling rate of most depth sensors is 30 Hz, whereas the 3DMC for gait analysis is more than 60 or 100 Hz^8^. Lower sampling rate might lead to a loss of important lower limb joint angle data.

Recently, RGB camera-based two-dimensional (2D) markerless human motion tracking systems, such as PoseNet^14^ and OpenPose (OP)^15^, have been developed to estimate human poses and body segments. OP is an open-source human pose estimation software that estimates human body keypoints using a two-branch multistage convolutional neural networks (CNN) from each RGB image as the input. Although these systems do not have depth sensors, they can estimate the human joint point using 2D images or videos with the CNN. These systems have great potential as gait analysis tools in clinical settings because they do not require the attachment of markers, technical skills, or immense costs. OP can be used as a screening tool for Parkinson’s disease^16^ and autism spectrum disorders^17^. There are some problems when using 2D pose estimation system for gait analysis. As pose estimation only estimate body keypoints, users are required to calculate joint kinematics from body segments and alignment. In addition, accuracy is required when OP is used for gait analysis^18^. Some previous study reported that OP can measure temporo-spatial and kinematic parameters in the sagittal plane during gait without substantial error^19,20^. Our previous study revealed that single RGB camera gait analysis by OP can measure several temporo-spatial parameters and sagittal plane joint angles with good to excellent agreement and consistency compared with optoelectronic 3DMC systems; however, the lower limb joint angle error increased in some gait conditions, including out of the 2D image plane excessive motion, such as increasing foot progression angle (FPA)^21^. In particular, the peak ankle joint angles had substantial error. When single RGB camera is used to measure a walking subject from the side, the 2D image plane of the camera captures flexion-extension motion easily. In contrast, out of the 2D image plane motion such as excessive FPA affect the accuracy of joint angle. The pose estimation algorithm also affected accuracy because this algorithm did not design for gait kinematics analysis in such conditions^22^. Although researchers attempted to decrease the joint angle errors caused by out of 2D image plane motion in single RGB camera analysis, the effective correction methods for out of 2D plane excessive motion and ankle joint angle error have been inadequately solved. Preparing many RGB cameras may decrease the angle error^23^; however, it is difficult to employ the measurement method using many cameras in clinical settings because of the large space and costs. 3D pose estimator using a single camera might be useful, however, this method has problems such as processing speed and ease of application when considering its use in clinical setting. To overcome these limitations, the complementation of OP kinematics using IMU data, such as segment acceleration and angular velocity, might be useful for improving accuracy.

In this study, we proposed a simple and accurate sensor-fusion approach for gait analysis using a single RGB camera and an IMU sensor. The primary aim of this study was to clarify whether the proposed method that fuses OP and IMU can decrease ankle joint angle error during gait. In the proposed method, an IMU was attached to foot segments to reduce the ankle joint angle error by using acceleration and angular velocity data. The secondary aim was to clarify temporo-spatial parameters and lower limb joint angles during four overground gait conditions with combinations of self-selected and slow speeds and with their normal FPA and with a larger FPA, as measured by 3DMC and OP. Although our previous study examined the accuracy of temporo-spatial parameters and joint kinematics by OP, we measured only treadmill gait^21^. Some previous studies have compared 3DMC and markerless gait analysis systems on overground gait^10,24^; however, the four gait conditions were not measured, including out of 2D image plane excessive motion, such as large FPA. Therefore, we measured temporo-spatial and kinematic parameters in the four gait conditions on the ground. Moreover, we used cross-correlation coefficients (CCC) for evaluating similarity of the data between these methods. We measured four gait conditions including large FPA conditions in healthy young adults using 3DMC and OP. To decrease the ankle angle error in the OP method, an IMU sensor data was fused with the OP data using a complementary filter. The 3DMC data served as the ground-truth labels.

## Methods

### Participants

Sixteen healthy adult males (age: 22 ± 1 years, height: 1.70 ± 0.05 m, mass: 64.4 ± 8.6 kg) participated in this study. The exclusion criteria were as follows: age < 20 years; limitation of physical activity owing to current injury or disease; history of lower limb surgery, neurological disorders, or cardiac disease; and back or leg pain during gait. All study procedures were approved by the ethics committee of Tokyo University of Science (20022), and written informed consent for study participation, publication of gait data, and images was obtained from all participants. This study was conducted in accordance with the principles of the Declaration of Helsinki.

### Motion task

The participants were randomly asked to walk under four conditions on a 9 m straight-line walkway: self-selected comfortable speed with normal and large FPA conditions, and slow speed with normal and large FPA conditions. The slow speed was set to 0.60 m/s, which was based on average of limited community ambulation in individuals after a stroke^25,26^. In normal FPA conditions, participants were asked to walk as usual. In large FPA conditions, the FPA of 50° was determined by a physical therapist during static position, and it was also checked during gait. The FPA in this study was greater than that in previous studies related to post-stroke and knee osteoarthritis gait^27–29^. The FPA was also monitored during gait using 3DMC. A large FPA is important for investigating the accuracy of motion analysis from 2D video images because this condition includes excessive motion in the 2D image plane. In addition, gait with a large FPA is often observed clinically. Although the self-selected speed with large FPA condition might not be commonly observed in clinical settings, we evaluated whether this condition for the OP could be measured in a difficult condition. Before the measurement, the participants had sufficient gait practice for each condition. Thereafter, gait trials were performed at least five times for each condition.

### Measurement procedures for 3DMC and OP

The OptiTrack motion capture system with 11 infrared cameras (NaturalPoint, Corvallis, OR, USA) were used for the ground-truth data of the 3DMC. A total of 31 markers were placed on anatomical landmarks. The markers were placed on the seventh cervical vertebra, sternoclavicular notch, xiphoid process, right scapular inferior angle, and the tenth thoracic vertebra. Additionally, the markers were affixed bilaterally over the acromion processes, anterior-superior and posterior-superior iliac spines, lateral thighs, medial and lateral epicondyles of the femurs, lateral shanks, medial and lateral malleoli, calcanei, head of second and fifth metatarsals, and tip of the second toes.

**Figure 1:**
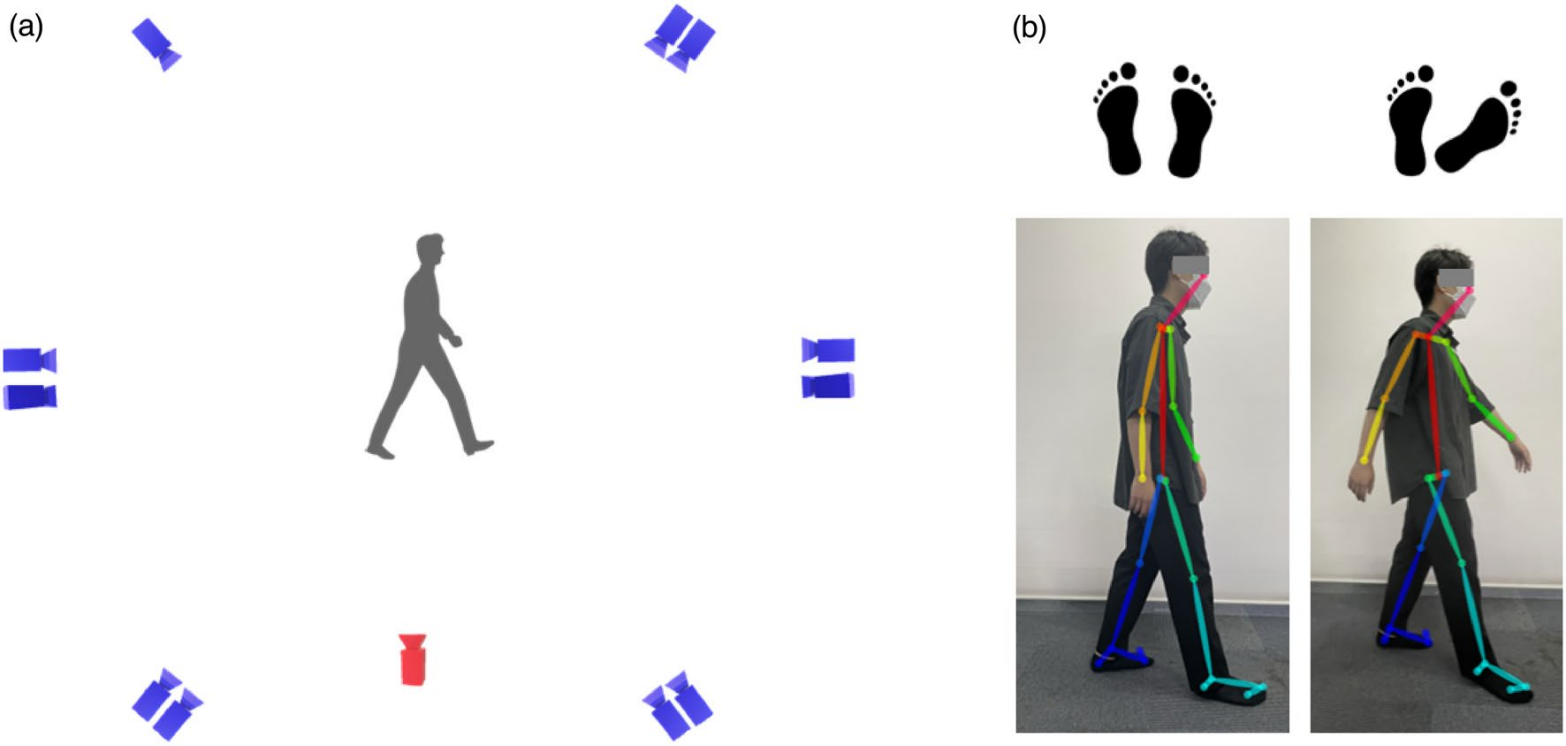
Overview of the experimental set up and body model of OP. (**a**) Approximate position of infrared cameras for 3DMC (blue) and an RGB camera for OP (red). (**b**) Body keypoints estimation by OP. BODY_25 model used in this study. Left side gait condition is normal foot progression angle (FPA), whereas right side is large FPA.

An RGB camera with a pixel resolution of 720 × 520 (Basler AG, Ahrensburg, Germany) was used for the 2D analysis using human pose estimation. This camera was positioned 3 m away from the participants, and it captured their dominant leg. The height of the camera was set at the greater trochanter of each participant. The RGB camera data were used for 2D pose estimation by OP. In the first stage, the part affinity fields, which form a set of 2D vector fields that encode the location and orientation of the extremities over the RGB images, were predicted^15^. In the second stage, the confidence maps of each human body segment location are predicted. Both stages were parsed to output 2D keypoints of the human body in the images. For the OP output, we used the Body_25 model, which provides the 2D coordinates of the 25 body keypoints in each RGB image (Fig. 1). More details on OP have been described by Cao et al.^15^. The data from 3DMC and OP were recorded at a sampling rate of 100 Hz, and 4th order low-pass Butterworth filter with a 6 Hz cutoff frequency was used.

### Data processing and proposed method for ankle joint angle

Temporo-spatial parameters, such as gait speed, stride length, stance time, and swing time were measured for each gait condition. In the 3DMC method, the midpoint of both posterior superior iliac spine (PSIS) markers was used to calculate the gait speed, whereas the gait speed measurement using the OP and the proposed method was calculated from the mean midhip keypoint velocity of the walkway direction in the 2D video. In both methods, initial contact/toe-off were defined based on the maximum or minimum point of antero-posterior distance between heel/second metatarsal head and midpoint of PSIS in 3DMC or heel/ big toe and midhip in OP by referring to a previous study^30^. Stride length was calculated from the distance between the initial and the next initial contact point of the dominant heel marker or keypoint coordination. In the OP method, the distance was calculated based on the length of tape on the walkway and number of pixels of the tape in the RGB camera image. In addition, representative kinematic parameters of peak joint angles for gait assessment were selected by referring to previous studies^31,32^. In the 3DMC method, flexion-extension angles of the lower limb joints were calculated using Skycom software (Accuity Inc., Tokyo, Japan). Hip, knee, and ankle joint flexion-extension angles in the OP method were calculated by body segment vectors. Keypoints for body segment vector were as follows: trunk, neck and midhip; thigh, hip and knee; shank, knee and ankle; foot, ankle and big toe. The hip angle was calculated from trunk and thigh segment vector. The knee angle was calculated from thigh and shank segment vectors. The ankle angle was calculated from shank and foot segment vectors. Joint angles in the OP method were calculated as follows:

1 $$angle={\mathrm{cos}}^{-1}\frac{\overrightarrow{{{\varvec{S}}}_{{\varvec{p}}}}\cdot \overrightarrow{{\boldsymbol{ }{\varvec{S}}}_{{\varvec{d}}}}}{\left|\overrightarrow{{{\varvec{S}}}_{{\varvec{p}}}}\right|\boldsymbol{ }\left|\overrightarrow{{{\varvec{S}}}_{{\varvec{d}}}}\right|}$$

where $\overrightarrow{{{\varvec{S}}}_{{\varvec{p}}}}$ and $\overrightarrow{{{\varvec{S}}}_{{\varvec{d}}}}$ are proximal and distal body segment vectors, respectively. Moreover, trailing limb angles (TLA) was also measured using both methods. TLA has been used as a useful outcome related to the forward propulsive force during gait^33–35^, and is defined as the angle between a vector joining the greater trochanter with the fifth metatarsal head and the laboratory’s vertical axis^32^. In the OP method, the TLA was calculated from the angle between the vector joining the dominant hip and small toe keypoints and a perpendicular line passing through center of the camera image. Knee joint angles obtained using OP methods tend to be underestimated by approximately 5°^21,36^; therefore, we compensated by adding 5° to the knee joint angle using OP methods.

**Figure 2:**
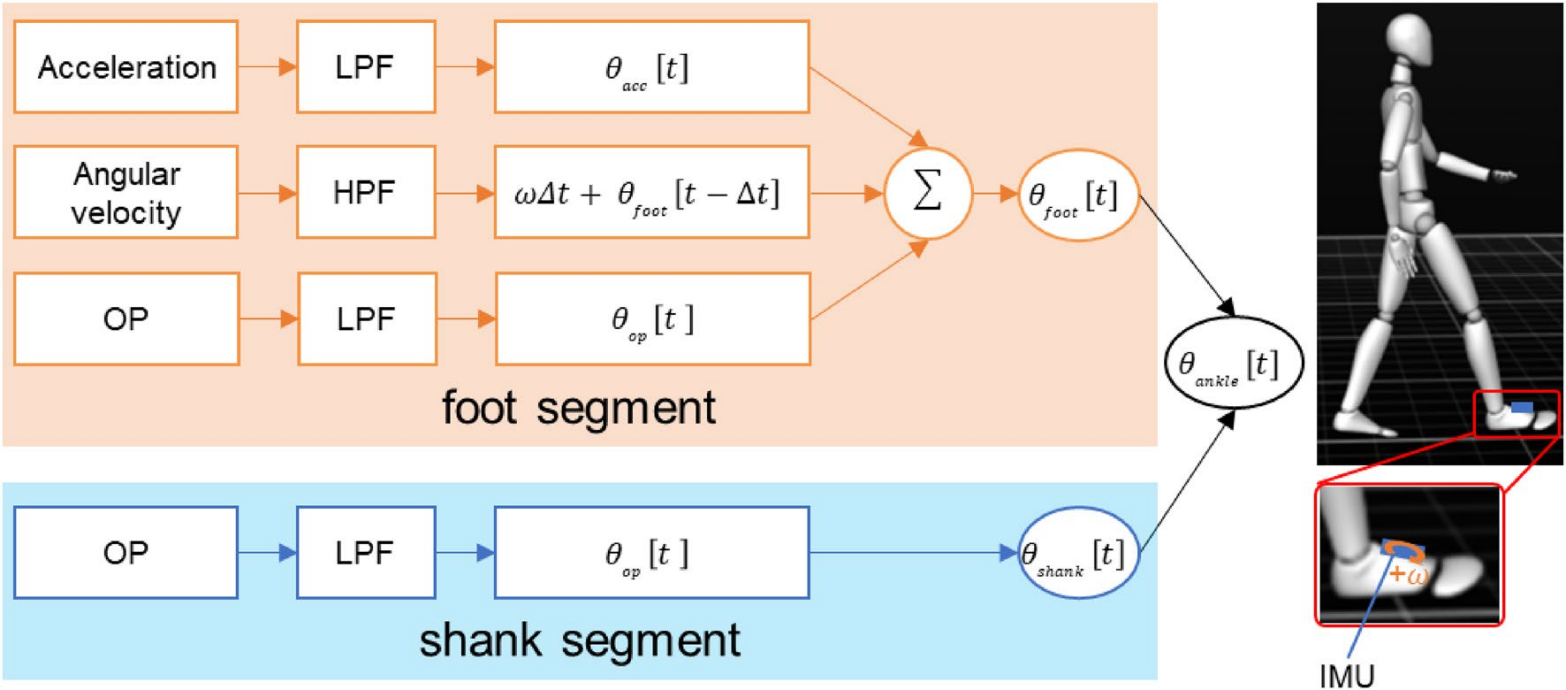
The proposed method fusing RGB camera-based pose estimation by OP and an IMU sensor on the foot segment to measure ankle joint angle. Foot segment angle was calculated from the acceleration, angular velocity, and OP data. LPF: low-pass filter; HPF: high-pass filter. A complementary filter was used to fuse the data based on each filter coefficients. Ankle joint angle was calculated using the fused foot segment angle and the shank segment angle from the OP.

To improve the accuracy of the ankle joint angle in the proposed method using OP, an IMU sensor (Delsys Inc., MA, USA) consisting of a triaxial accelerometer and gyroscope was attached to the participants’ dominant foot segment. The sampling rate of the IMU was 148 Hz. The acceleration and angular velocity data were filtered by a low-pass filter with a 6 Hz cutoff frequency and high-pass filter with a 0.5 Hz cutoff frequency. RGB camera, 3DMC and IMU were synchronized by an electrical trigger. In the proposed method, a complementary filter was used to fuse the OP, acceleration, and angular velocity of the foot segment angle in the sagittal plane (Fig. 2). The complementary filter can simply and accurately calculate the joint angle during gait^37,38^. The foot segment angle in the sagittal plane (${\theta }_{foot}$) applied to the filter was calculated as follows:

2 $${\theta }_{foot}\left[t\right]=B{\theta }_{a}\left[t\right] + C\left( {\theta }_{foot} \left[t - \Delta t\right]+ \omega \left[\mathrm{t}\right]\Delta t\right)+ \left(1 - B - C\right){\theta }_{OP}\left[t\right]$$

where $t$ is the time; ${\theta }_{a}$ and ${\theta }_{OP}$ are the foot segment angles calculated from the acceleration and OP data, respectively. $\omega$ is the filtered foot segment angular velocity obtained from the gyroscope. $B$ and $C$ are the filter coefficients. ${\theta }_{a}$ was calculated using the filtered acceleration via a trigonometric operation. The filter coefficients under normal and large FPA conditions were set from empirical tests based on a previous study^37^. In this study, normal FPA conditions of $B$ and $C$ were 0.02 and 0.85, respectively, while the corresponding large FPA conditions were 0.05 and 0.77, respectively. The ankle dorsiflexion and plantarflexion angles of the proposed methods were calculated using the foot segment angle applied to the complementary filter and the shank segment angle from the OP. As an index of measurement parameters, the temporo-spatial parameters, peak hip and peak knee flexion–extension angles, and TLA using the 3DMC and OP method were compared for each condition. Because the knee joint angle during a gait cycle has peak flexion in the early and late stance phases, both peak flexions were measured as 1st and 2nd peak flexion angles. Peak dorsiflexion and plantarflexion angles using the 3DMC, OP, and the proposed method were compared for each condition. The Mean absolute errors (MAE) of the temporo-spatial and kinematic parameters between the 3DMC and OP or the proposed methods were calculated.

### Statistical analysis

For each parameter, the assumption of normality was assessed using the Shapiro–Wilk test. The MAE between the 3DMC and OP methods for temporo-spatial parameters and kinematic parameters among the four gait conditions was assessed by a one-way repeated measures analysis of variance, followed by Shaffer’s modified sequentially rejective Bonferroni procedure as a post hoc test. In addition, the MAE between the 3DMC and the proposed methods for peak ankle joint angles was compared using the same statistical analysis. To verify the effectiveness of the proposed method on the ankle joint angle, a two-tailed paired *t*-test was used to compare the MAE obtained by OP with the MAE obtained by the proposed method. The Friedman and Wilcoxon signed-rank tests were used to compare data without normality. Furthermore, CCC between these methods was used to evaluate the similarity of angles during the gait cycle. The CCC values were interpreted as weak or no coupling (−0.3 < CCC < 0.3), moderate coupling (0.3 ≤ CCC < 0.70 or −0.7 < CCC ≤ −0.3), and strong coupling (CCC > 0.7 or CCC < −0.7)^39^. Statistical significance was set at *p* < 0.05.

## Results

The mean ± standard deviation (SD) of temporo-spatial parameters, hip kinematic parameters, and knee kinematic parameters under the four conditions are listed in Tables 1 and 2. The OP and proposed methods employed the same calculations for these parameters and so only OP results are shown in Tables 1 and 2. The ankle joint angles of the 3DMC, OP, and proposed methods under the four conditions are listed in Tables 3 and 4. MAE were averaged after calculating the absolute value of the difference between each participant’s data measured by 3DMC and OP or the proposed methods. MAE of lower limb joint angle between the methods was shown in Fig. s1 to s3 (see supplementary material).

**Table 1:** Temporo-spatial parameters in the four gait conditions (mean ± SD).

		Self-selected speed			Slow speed		
		3DMC	OP	MAE	3DMC	OP	MAE
Gait speed (m/s)	Normal	1.05 ± 0.16	1.06 ± 0.17	0.02 ± 0.03	0.67 ± 0.12	0.67 ± 0.12	0.01 ± 0.01
	Large FPA	0.89 ± 0.16	0.91 ± 0.17	0.02 ± 0.02	0.62 ± 0.12	0.63 ± 0.12	0.02 ± 0.02
Stride length (m)	Normal	1.22 ± 0.10	1.24 ± 0.11	0.03 ± 0.04	0.94 ± 0.14	0.94 ± 0.14	0.04 ± 0.05
	Large FPA	1.09 ± 0.16	1.11 ± 0.16	0.06 ± 0.06	0.88 ± 0.16	0.91 ± 0.17	0.04 ± 0.04
Stance time (s)	Normal	0.76 ± 0.11	0.77 ± 0.11	0.01 ± 0.02	0.98 ± 0.18	0.98 ± 0.19	0.01 ± 0.02
	Large FPA	0.80 ± 0.12	0.80 ± 0.12	0.01 ± 0.01	0.97 ± 0.19	0.96 ± 0.20	0.01 ± 0.01
Swing time (s)	Normal	0.40 ± 0.04	0.40 ± 0.04	0.01 ± 0.01	0.45 ± 0.06	0.43 ± 0.07	0.02 ± 0.03
	Large FPA	0.42 ± 0.04	0.41 ± 0.06	0.02 ± 0.03	0.45 ± 0.06	0.45 ± 0.06	0.02 ± 0.02

In large FPA conditions, participants were instructed to walk with an FPA of 50°. OP and proposed methods used the same calculation. MAE was averaged after calculating the absolute value of the difference between each participant’s data measured by two methods.

**Table 2:** Peak hip and knee joint angles during a gait cycle in the four gait conditions (mean ± SD).

(deg)		Self-selected speed			Slow speed		
		3DMC	OP	MAE	3DMC	OP	MAE
Hip flexion	Normal	22.4 ± 3.6	23.4 ± 2.8	3.1 ± 1.7	19.4 ± 3.2	20.1 ± 2.9	2.4 ± 1.8
	Large FPA	21.7 ± 5.1	25.1 ± 4.7	4.5 ± 3.1	19.1 ± 6.4	21.9 ± 4.3	4.0 ± 3.5
Hip extension	Normal	19.3 ± 5.0	18.1 ± 2.7	4.1 ± 2.4	14.1 ± 4.2	15.2 ± 3.6	3.5 ± 2.3
	Large FPA	18.5 ± 4.8	14.3 ± 4.8	4.8 ± 3.3	14.3 ± 4.9	11.8 ± 4.8	4.2 ± 3.4
TLA	Normal	29.9 ± 2.8	29.4 ± 2.4	1.6 ± 1.3	26.5 ± 2.7	26.0 ± 2.9	1.9 ± 1.5
	Large FPA	25.3 ± 3.6	27.0 ± 3.4	3.0 ± 1.6	22.9 ± 4.3	24.5 ± 4.3	2.5 ± 1.5
Knee flexion (1st)	Normal	24.7 ± 5.1	21.6 ± 5.4	4.7 ± 2.3	19.6 ± 6.9	17.4 ± 4.7	4.4 ± 2.6
	Large FPA	22.6 ± 8.4	20.2 ± 6.8	4.9 ± 2.9	18.3 ± 9.6	16.7 ± 6.9	5.0 ± 3.1
Knee flexion (2nd)	Normal	68.1 ± 4.3	66.6 ± 5.2	2.4 ± 1.9	62.7 ± 4.3	61.6 ± 4.3	2.2 ± 1.7
	Large FPA	55.4 ± 7.9	54.1 ± 7.6	2.3 ± 1.8	48.2 ± 9.6	46.8 ± 9.4	3.5 ± 2.4
Knee extension	Normal	9.2 ± 4.3	7.5 ± 2.3	3.7 ± 2.3	8.5 ± 4.7	7.7 ± 2.6	3.3 ± 2.2
	Large FPA	8.4 ± 6.2	9.0 ± 4.1	3.9 ± 2.3	7.6 ± 6.2	9.1 ± 4.8	4.1 ± 2.5

In large FPA condition, participants were instructed to walk with an FPA of 50°. OP and proposed methods used the same calculation. MAE was averaged after calculating the absolute value of the difference between each participant’s data measured by two methods.

**Table 3:** Peak ankle joint angles during a gait cycle calculated by 3DMC, OP, and proposed methods in self-selected speed conditions (mean ± SD).

(deg)		3DMC	OP	Proposed	MAE |3DMC—OP|	MAE |3DMC—Proposed|
Dorsiflexion	Normal	18.9 ± 3.6	11.7 ± 3.5	19.3 ± 3.7	7.4 ± 3.7 * *p* < 0.001 † *p* = 0.005	3.2 ± 2.7
	Large FPA	19.8 ± 3.2	7.9 ± 5.9	24.7 ± 4.5	12.3 ± 5.2	5.5 ± 3.9
Plantarflexion	Normal	8.0 ± 5.9	10.9 ± 4.4	10.8 ± 4.4	5.2 ± 3.8 * *p* < 0.001 † *p* < 0.001	5.1 ± 3.6 * *p* < 0.001
	Large FPA	−2.1 ± 3.1	15.2 ± 6.3	8.9 ± 6.1	17.3 ± 6.1	11.1 ± 5.7

In large FPA condition, participants were instructed to walk with an FPA of 50°. MAE was averaged after calculating the absolute value of the difference between each participant’s data measured by two methods. *Indicates a significant difference compared to self-selected speed with large FPA condition on same MAE (*p* < 0.05). † indicates a significant difference compared to slow speed with large FPA condition on same MAE (*p* < 0.05).

**Table 4:** Peak ankle joint angles during a gait cycle calculated by 3DMC, OP, and proposed methods in slow speed conditions (mean ± SD). In large FPA condition, participants were instructed to walk with an FPA of 50°.

(deg)		3DMC	OP	Proposed	MAE |3DMC—OP|	MAE |3DMC—Proposed|
Dorsiflexio	Normal	19.5 ± 3.4	11.6 ± 3.5	19.9 ± 3.5	7.9 ± 4.3 * *p* = 0.005 † *p* = 0.016	3.5 ± 2.5
	Large FPA	18.7 ± 3.7	6.3 ± 4.9	21.7 ± 5.2	12.4 ± 5.8	5.3 ± 3.9
Plantarflexion	Normal	2.7 ± 4.7	9.3 ± 4.2	7.1 ± 4.8	6.7 ± 4.1 * *p* < 0.001 † *p* < 0.001	4.5 ± 3.6 * *p* < 0.001 † *p* = 0.008
	Large FPA	−0.4 ± 5.6	15.8 ± 7.3	7.5 ± 6.9	16.5 ± 7.9	8.7 ± 6.3

MAE was averaged after calculating the absolute value of the difference between each participant’s data measured by two methods. *Indicates a significant difference compared to self-selected speed with large FPA condition on same MAE (*p* < 0.05). † indicates a significant difference compared to slow speed with large FPA condition on same MAE (*p* < 0.05).

The MAE in gait speed and stride length under the four conditions were up to 0.02 m/s and 0.06 m, respectively. The MAE in stance and swing times were up to 0.02 s (two motion capture frames). Most of the MAE values for these parameters were less than 5% of the 3DMC values. The MAE of gait speed, stride length, stance time, and swing time were not significantly different among the four conditions (*p* = 0.290, *p* = 0.248, *p* = 0.547, and *p* = 0.432, respectively).

**Figure 3:**
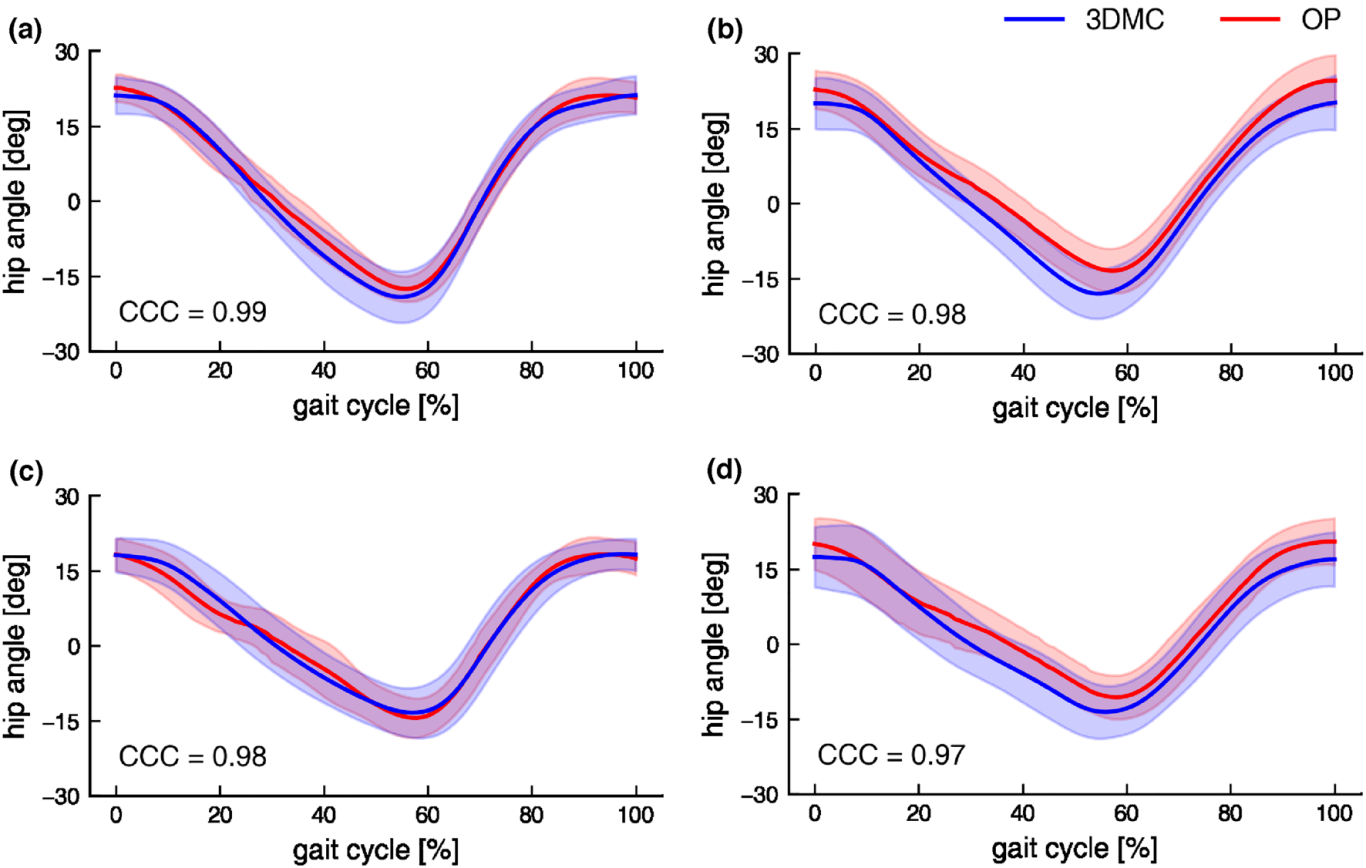
Hip angle by the two measurement methods. Self-selected speed with normal (**a**) and large FPA (**b**) condition. Slow speed with normal (**c**) and large FPA (**d**) condition. The shade is presented as 1 SD. Flexion is defined as positive.

**Figure 4:**
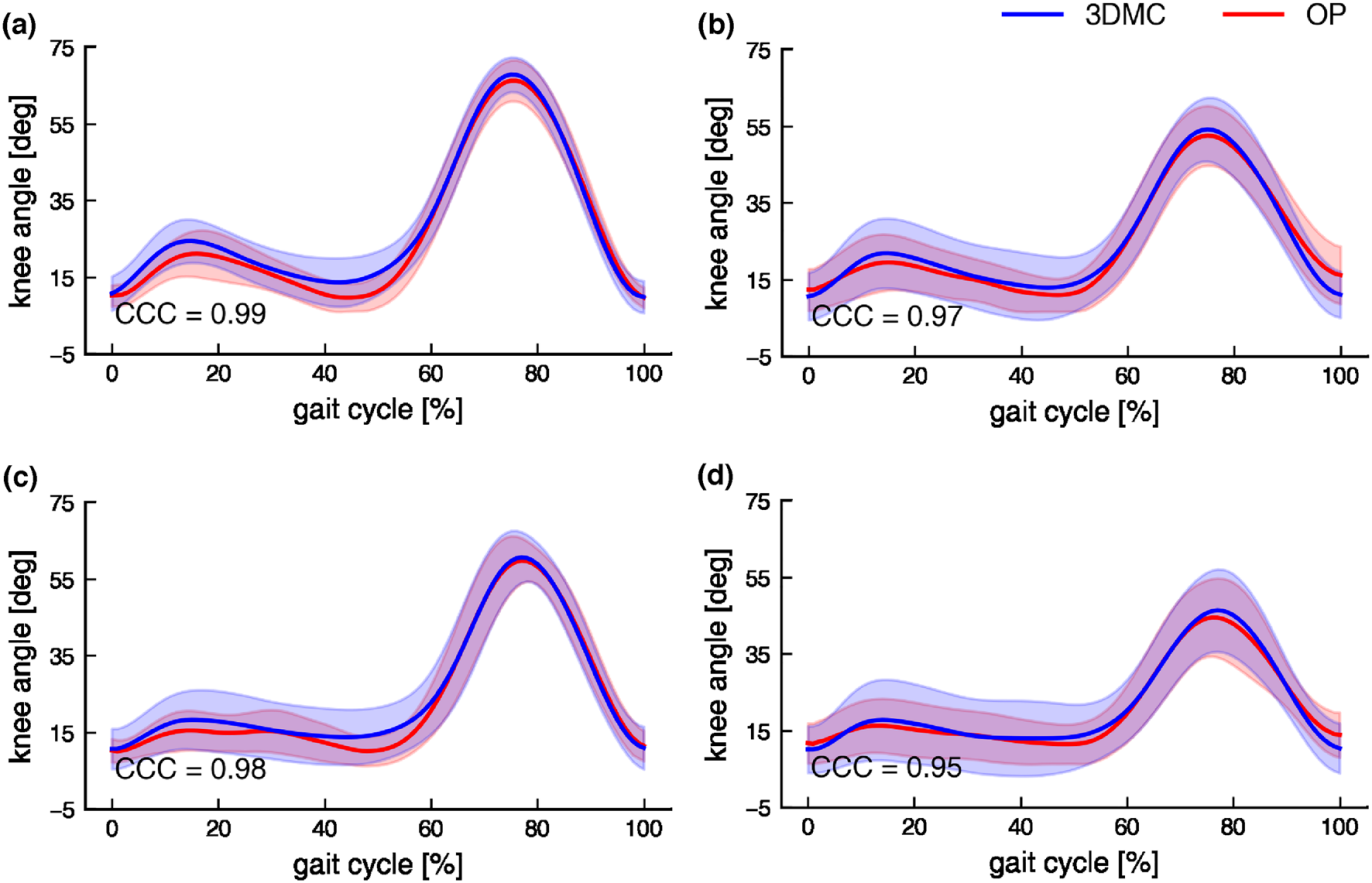
Knee angle by the two measurement methods. Self-selected speed with normal (**a**) and large FPA (**b**) condition. Slow speed with normal (**c**) and large FPA (**d**) condition. The shade is presented as 1 SD. Flexion is defined as positive.

The hip and knee joint angles in the sagittal plane of temporal changes during gait under the four conditions are shown in Figs. 3 and 4. Most of the MAE for hip and knee peak kinematic parameters in each condition were less than 5° (Table 2). The MAE of the peak hip flexion angle and TLA differed significantly among the four conditions (both *p* = 0.040). Nevertheless, the post-hoc test results did not vary significantly. The MAE of the peak hip extension angle was not significantly different among the four conditions (*p* = 0.475). In addition, CCC of the hip and knee joint angles between the 3DMC and OP methods were strongly coupled in each condition (Figs. 3 and 4).

**Figure 5:**
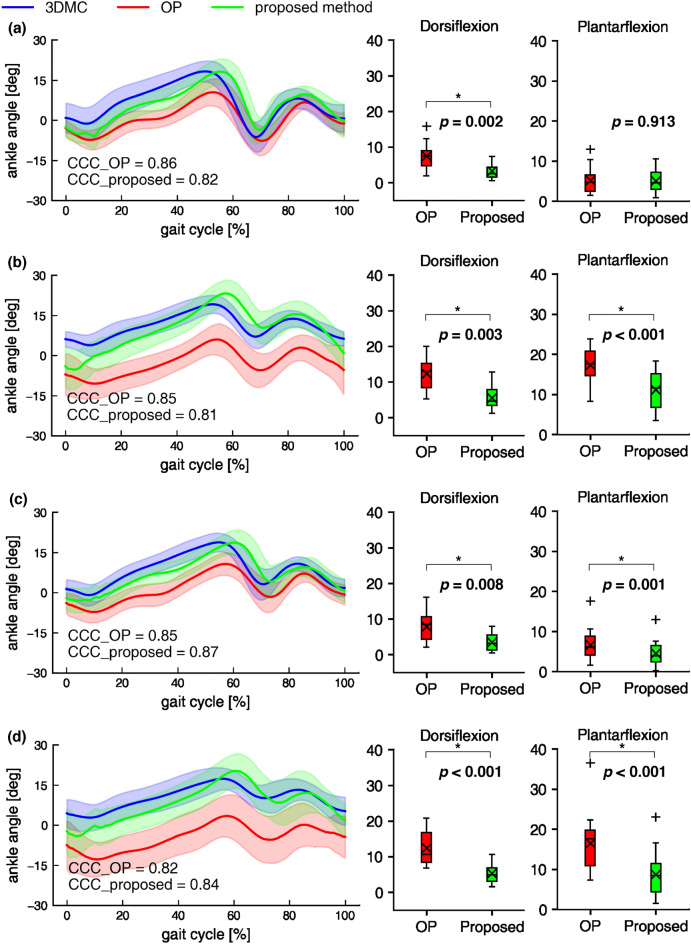
Ankle dorsiflexion-plantarflexion angle by the three measurement methods and MAE of peak joint angles in each condition. Left side figures show ankle joint angles of temporal changes during gait under the four conditions. Self-selected speed with normal (**a**) and large FPA (**b**) condition. Slow speed with normal (**c**) and large FPA (**d**) condition. The shade is presented as 1 SD. Flexion is defined as positive. The right side box plots show MAE between 3DMC and other methods. Red and green box plots mean MAE using OP and proposed method, respectively. Boxes and horizontal lines represent ranges of Q1–Q3 and median values. × and + indicates mean and outlier, respectively. *Indicates that proposed method significantly decreased MAE compared with MAE using OP (*p* < 0.05).

Ankle joint angles in the sagittal plane of temporal changes during gait calculated by 3DMC, OP, and the proposed method are shown in Figs. 5. The OP method had substantial peak ankle angle errors under large FPA conditions. In obtaining the MAE between the 3DMC and OP methods, the peak dorsiflexion and plantarflexion angles significantly differed among the four conditions (both *p* < 0.001). The post hoc test identified that the peak dorsiflexion angle in the self-selected and slow speeds with normal FPA condition significantly decreased compared to that of the self-selected and slow speeds with large FPA condition (Tables 3 and 4). The MAE between the 3DMC and OP methods of the peak plantarflexion angle in self-selected and slow speeds with normal FPA conditions also significantly decreased compared to that of the self-selected and slow speeds with large FPA conditions.

Comparison of the ankle joint angle MAE using the OP and using the proposed methods are shown in Fig. 5. The proposed method significantly decreased the MAE of peak dorsiflexion angle compared with the OP method under all conditions (lowest *p* < 0.001). The MAE of peak plantarflexion angles was significantly decreased in the proposed method under all conditions, except for the self-selected speed with the normal FPA condition (lowest *p* < 0.001). Moreover, the CCC of the ankle joint angles to 3DMC in OP and the proposed method showed strong coupling under all conditions (Fig. 4). In the MAE between 3DMC and the proposed method, although the peak dorsiflexion angle significantly differed among the four conditions (*p* = 0.009), no statistically significant difference was obtained by the post hoc test (lowest *p* = 0.118). The peak plantarflexion angle in MAE between the 3DMC and the proposed method significantly differed among the four conditions (*p* < 0.001). The post hoc test identified that the peak plantarflexion angle in the slow speed with normal FPA condition significantly decreased compared to the self-selected and slow speed with large FPA condition (Tables 3 and 4).

## Discussion

The primary aim of this study was to clarify the capability of the gait analysis method fusing RGB camera-based pose estimation and an IMU sensor on the foot segment to measure ankle joint angle during gait. In addition, this study compared temporo-spatial parameters and lower limb joint angles during overground gait under various conditions, as measured by the 3DMC and RGB camera-based pose estimation system. The participants walked under four conditions with different gait speeds and FPA. Temporo-spatial parameters and peak lower limb joint angles during the four gait conditions were measured using 3DMC and OP. The peak ankle joint angles were compared among the three methods, including the proposed method. In the proposed method, an IMU sensor was attached to the foot segment, and its acceleration and angular velocity data were fused to the OP to decrease the ankle angle error.

The MAE of temporo-spatial parameters, such as gait speed, stride length, stance time, and swing time, were not statistically significant among the four overground gait conditions. In this study, we examined large FPA gait conditions because these conditions, including out of 2D image plane excessive motion, affect the measurement of temporo-spatial parameters and joint angle by OP. The results showed that the MAE between 3DMC and OP of these parameters under all conditions was less than 5% of the 3DMC values. The highest MAE values of gait speed, stride length, stance time, and swing time among the four conditions were 0.02 m/s, 0.06 m, 0.01 s, and 0.02 s, respectively. The MAE of temporo-spatial parameters was less than the minimal detectable change in healthy and post-stroke gait using 3DMC in test-retest experiments^40,41^. In addition, these temporo-spatial parameters obtained using OP among the four conditions were similar or improved compared to those observed in previous studies conducted using the markerless gait analysis system^13,42^. These results might be attributed to the high sampling rate. The sampling rate of this study was 100 Hz, which is comparable to that of the optoelectronic 3DMC. Therefore, OP could accurately measure the temporo-spatial parameters under various overground gait conditions by detecting heel contact and toe-off accurately.

The MAE of the peak hip and knee joint angles in the sagittal plane was also not statistically significant among the four overground gait conditions. All the MAE between the 3DMC and OP of the peak hip and knee joint angles were less than 5° of the 3DMC values except for 1st peak knee flexion angle in slow speed with large FPA condition. The highest MAE of the peak hip joint angles, TLA, and peak knee joint angles were 4.8°, 3.0°, and 5.0°, respectively. Although the MAE of the hip, knee, and TLA tended to increase under large FPA conditions, the MAE was less than the minimal detectable change in healthy and post-stroke gait using 3DMC in test-retest experiments^40,41^. These errors in hip and knee joint kinematics parameters between 3DMC and OP in all conditions were similar or less than those in previous studies using OP on normal FPA gait conditions^20,36^. Moreover, the CCC of the hip and knee joint angles on the sagittal plane of temporal changes in between 3DMC and OP were strongly coupled (CCC > 0.70) in all conditions. These results might be attributed to the high sampling rate (100 Hz). Furthermore, accurate body keypoint detection of OP using two multistage CNN might contribute to these results^15^. Therefore, OP has the potential to measure hip and knee joint angle parameters under various overground gait conditions, as well as 3DMC. However, note that OP might not accurately measure these angles, such as gait with larger FPA or more excessive rotation compared with this study, because of excessive motion affection in the 2D image plane.

In the MAE between the 3DMC and OP methods, peak dorsiflexion and plantarflexion angles of the sagittal plane were statistically significant among the four overground gait conditions, although the CCC of ankle joint angles of temporal changes showed strong coupling (CCC > 0.70) in all conditions. The MAE between 3DMC and OP of peak joint angles was large compared with hip and knee joint angles. The MAE of peak joint angles under large FPA conditions was more than 10°. These results are consistent with those of previous studies that used a markerless gait analysis system^36,43^. The large error in the ankle joint angle might have been caused by the smaller foot segment and faster ankle angular velocity.

The proposed method significantly decreased the MAE of peak dorsiflexion and plantarflexion angle compared with the OP method under all conditions, except for the MAE of plantarflexion at a self-selected speed with the normal FPA condition (Fig. 5). Moreover, most of MAE between 3DMC and the proposed method on the peak ankle joint angle were less than the minimal detectable change in post-stroke gait using 3DMC in test-retest experiments^40,41^. CCC of ankle joint angles in the proposed method showed strong coupling (CCC > 0.70) in all conditions. Because the proposed method is fused to the OP and an IMU sensor on the foot segment, this method seemingly improves foot segment angle by compensating for the acceleration and angular velocity data of the IMU. However, only peak plantarflexion angles of the sagittal plane were statistically significant among the four overground gait conditions in MAE between the 3DMC and the proposed method. This difference may be caused by the large MAE under large FPA conditions. The results of our study showed that the peak plantarflexion angle by the OP method had a substantial error compared with 3DMC, especially in large FPA conditions. Although the proposed method significantly decreased the MAE of the peak plantarflexion angle, the MAE at a slow speed with a large FPA condition was still 8.7°. Even markerless gait analysis systems with multiple RGB cameras have substantial errors in the plantarflexion angle^23^. The complementary filter used in this study might not have been sufficiently compensated because the plantarflexion angle calculated by pose estimation had a large error under large FPA conditions. Therefore, although the proposed method can measure the ankle angle during gait, we should note that a large FPA condition causes an increase in plantarflexion angle error.

This study has a few limitations. First, the participants were young, healthy adults. Although we measured various gait conditions in this study, future studies should include participants with gait disabilities. Second, we did not evaluate the effect of keypoint estimation accuracy on wearing loose clothing, using assistive devices or shoes, and changes in lighting. These changes may affect the accuracy of body keypoint estimation. Finally, pose estimation is based on musculoskeletal alignment. Wearing large assist devices or prostheses may result in misestimation of keypoints. Despite these limitations, the proposed method fusing OP and an IMU sensor has the potential to measure temporo-spatial parameters and lower limb joint kinematics in the sagittal plane in various gait patterns.

In conclusion, we aimed to clarify the capability of the gait analysis method fusing RGB camera-based pose estimation by OP and an IMU sensor on the foot segment to measure ankle joint kinematics during various gait conditions. We compared temporo-spatial parameters and lower limb joint angles during overground gait under various conditions, as measured by the 3DMC and OP. The results indicated that the proposed methods could measure temporo-spatial parameters and lower limb joint angles in the sagittal plane under various gait conditions. Future studies should include measurements of patients with gait disabilities to verify the practicality of this method in clinical settings.

## Supplementary Information

Supplementary Information.

## Data Availability

The data generated during this study are not publicly available due to ethical restriction regarding subjects’ personal information but are partially available from the corresponding author upon reasonable request.
