# Supplementary material for: Verification of gait analysis method fusing camera-based pose estimation and an IMU sensor in various gait conditions
Source: Sci Rep. 2022 Oct 21;12:17719. doi: 10.1038/s41598-022-22246-5 (PMC9586966; doi:10.1038/s41598-022-22246-5)
Supplement: Supplementary file 1 — Supplementary Information. [file 41598_2022_22246_MOESM1_ESM.pdf]

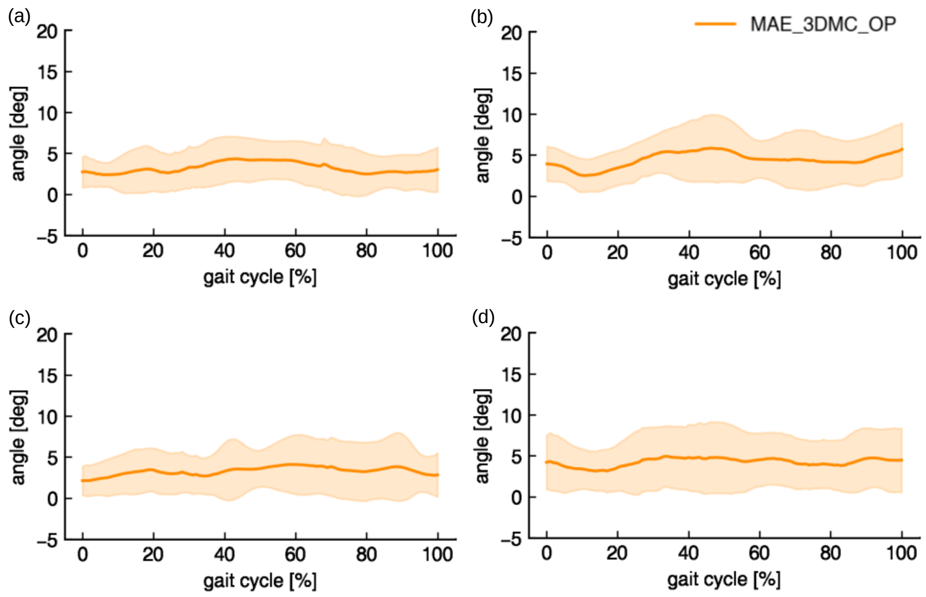

Figure s1. MAE of hip angle by the two measurement methods Self-selected speed with normal (a) and large FPA (b) condition. Slow speed with normal (c) and large FPA (d) condition. The shade is presented as 1 SD. Flexion is defined as positive.

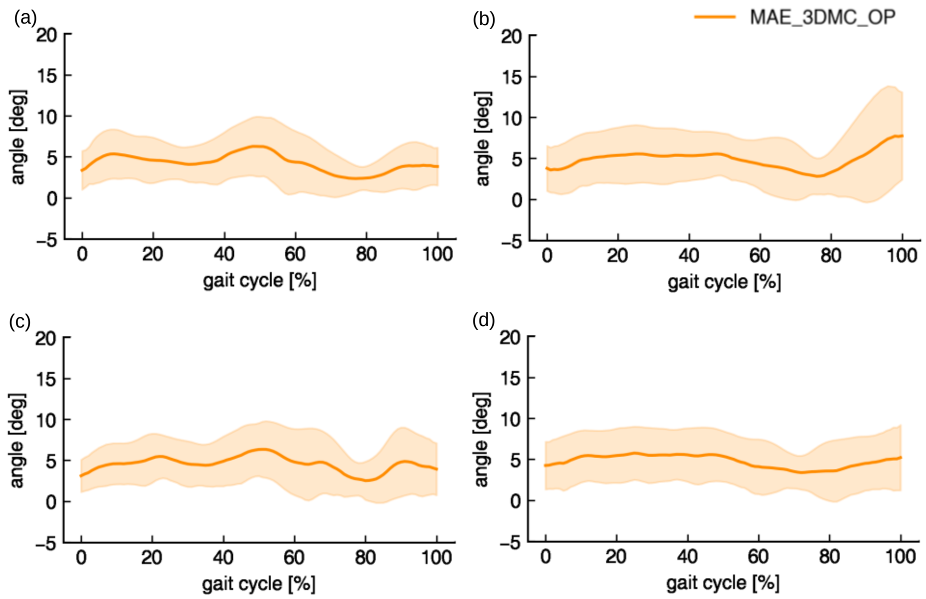

Figure s2. MAE of knee angle by the two measurement methods Self-selected speed with normal (a) and large FPA (b) condition. Slow speed with normal (c) and large FPA (d) condition. The shade is presented as 1 SD. Flexion is defined as positive.

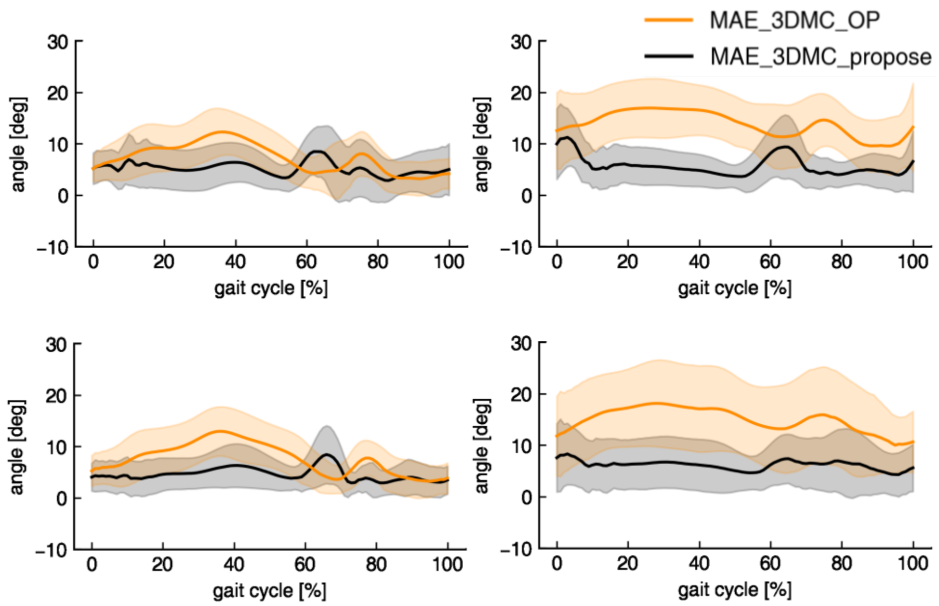

Figure s3. MAE of ankle angle between 3DMC and other methods Self-selected speed with normal (a) and large FPA (b) condition. Slow speed with normal (c) and large FPA (d) condition. The shade is presented as 1 SD. Flexion is defined as positive.
